# The Effect of Interactivity, Tailoring, and Use Intensity on the Effectiveness of an Internet-Based Smoking Cessation Intervention Over a 12-Month Period: Randomized Controlled Trial

**DOI:** 10.2196/47463

**Published:** 2023-11-21

**Authors:** Phillip Maiwald, Martina Bischoff, Peter Lindinger, Iris Tinsel, Matthias Sehlbrede, Urs Alexander Fichtner, Gloria Metzner, Christian Schlett, Erik Farin-Glattacker

**Affiliations:** 1 Institute of Medical Biometry and Statistics, Section of Health Care Research and Rehabilitation Research Faculty of Medicine and Medical Center University of Freiburg Freiburg Germany; 2 Wissenschaftlicher Arbeitskreis Tabakentwöhnung (WAT) e V Universitätsklinik für Psychiatrie und Psychotherapie Tübingen Germany

**Keywords:** eHealth, internet-based, smoking cessation, interactivity, tailoring, use intensity, randomized controlled trial, mobile phone

## Abstract

**Background:**

eHealth approaches show promising results for smoking cessation (SC). They can improve quit rates, but rigorous research is sparse regarding their effectiveness and the effects of their interactivity, tailoring, and use intensity.

**Objective:**

We examined the effectiveness of *Techniker Krankenkasse Smoking Cessation Coaching (TK-SCC),* an internet-based, tailored, and interactive SC intervention. Our hypotheses were as follows: hypothesis 1, in the intervention group (IG; access to TK-SCC), a clinically relevant number of participants will be abstinent at the 12-month follow-up (T3); hypothesis 2, the number of abstinent participants will be significantly greater in the IG than the control group (CG) at T3; and hypothesis 3, in the IG, more intense use of TK-SCC will be positively associated with abstinence.

**Methods:**

Individuals who smoke were randomized into the IG (563/1115, 50.49%) or CG (552/1115, 49.51%), which received a noninteractive, nontailored, and information-only web-based intervention. Data were collected before the intervention, at the postintervention time point (T1), at the 4-month follow-up (T2), and at T3. We tested hypothesis 1 through equivalence tests between the IG’s success rate and success rates of comparable effective interventions reported in 2 current meta-analyses. For hypothesis 2, we conducted binary logistic regressions. For hypothesis 3, we assigned the IG participants to 1 of 4 user types and used binary logistic regressions with user types as the independent variable and smoking abstinence as the dependent variable.

**Results:**

In the IG, 11.5% (65/563) and 11.9% (67/563) of participants were smoke free at T1 and T3, respectively. These values were statistically equivalent to the effects in the 2 meta-analyses, which reported 9% (*z* score=0.64, *P*=.74) and 10.9% (*z* score=−0.71, *P*=.24) success rates, respectively. In the CG, 6.2% (34/552) of the participants were smoke free at T1, which increased up to 8.2% (45/552) at T3. The difference between the IG and CG was statistically significant only at T1 (odds ratio [OR] 2.0, 99% CI 1.1 to 3.6; *P*=.002), whereas the effect was nonsignificant following α error corrections at T3 (OR 1.6, 99% CI 0.9 to 2.7; *P*=.02). In the IG, constant users of the program became smoke free significantly more often than rare users of the program (T1: OR 15.0, 99% CI 6.1 to 36.9; *P*<.001; T3: OR 6.5, 99% CI 2.8 to 15.5; *P*<.001).

**Conclusions:**

TK-SCC is effective for SC. However, its superiority compared with a minimal SC intervention could not be confirmed in the long term. Insufficient implementation of the techniques used and cotreatment bias could explain this outcome. Higher use intensity of TK-SCC was positively related to abstinence. Therefore, additional efforts to motivate users to adhere to intervention use as intended could improve the intervention’s effectiveness.

**Trial Registration:**

German Clinical Trials Register DRKS00020249, Universal Trial Number U1111-1245-0273; https://drks.de/search/de/trial/DRKS00020249

**International Registered Report Identifier (IRRID):**

RR2-10.1186/s13063-021-05470-8

## Introduction

### Background

Smoking cessation (SC) reduces the risk of premature death by 40% if individuals who smoke quit before 60 years of age and by 90% if individuals who smoke quit before 40 years of age [1,2]. Therefore, a reduction in smoking prevalence is a highly desirable prevention goal. However, the vast majority of quit attempts are made unassisted [3,4], although this is rarely effective (only 3% to 5% success rate in the long term [5]). To date, the most effective SC intervention consists of counseling in combination with medication [6,7], but this treatment is not accessible for many individuals who smoke because of various barriers, such as a lack of support to quit from health and other service providers [8,9]. Therefore, innovative ways to provide accessible support for individuals who are trying to quit smoking are needed.

eHealth approaches are highly promising because they provide a readily available, low-cost treatment option. eHealth uses electronic communication and information technologies to promote health behaviors, behavior changes, and psychoeducation [10]. Such interventions are capable of reaching a large number of individuals who smoke who would otherwise try quitting unassisted [11], and their effectiveness has been demonstrated in the short term as well as long term [12]; however, the grade of evidence is often low, and results can be mixed, depending on which kinds of interventions are compared [13,14]. Apparently, eHealth programs for SC can be effective tools for improving quit rates among individuals who smoke, but so far, the level of evidence is low.

Possible ways to increase the effectiveness of eHealth interventions include the incorporation of interactivity and tailoring (personalization of an intervention). In a recent meta-analysis, tailored or interactive internet interventions were not found to be superior compared with other internet interventions [14]. Similarly, another meta-analysis did not find significant effects when internet-based SC interventions that were either interactive or tailored were compared with a control group (CG) [15]. However, the former publication reported that tailored and interactive internet-based SC interventions are more effective than nonactive control interventions and that tailored messages are more effective than nontailored messages [14]. As a result, the evidence concerning the effectiveness of interactivity and tailoring remains inconclusive, and the most promising approach seems to be the combination of both techniques. Concerning the mode of action, the effects of tailoring can, at least in part, be attributed to higher user adherence [16], and more intense use of SC programs has been shown to be related to higher quitting success [17,18].

Overall, the demand for rigorous randomized controlled trials (RCTs) of eHealth interventions for SC is high. In particular, evidence regarding the effectiveness of given interventions, evidence of the effects of interactivity and tailoring, and evidence of the effect of use intensity are needed.

### Purpose and Hypotheses

The purpose of the research presented in this paper was to examine the effectiveness of a tailored and interactive internet-based intervention (intervention group [IG]) compared with a noninteractive, nontailored, and information-only internet-based intervention (CG) for SC in a sample of individuals who smoke. In the IG, we expected a clinically relevant number of participants to remain abstinent at the 12-month follow-up (T3; hypothesis 1). Furthermore, we assumed the number of abstinent participants will be significantly greater in the IG than in the CG after 1 year (hypothesis 2). In addition, we predicted that a higher dose (a more intense use of the internet-based health coach) will be positively related to a higher number of successful quitting attempts in the IG (hypothesis 3).

## Methods

### Trial Background

Our study was part of a larger project aiming to evaluate an internet-based health coach [19] with regard to increased physical activity, sustainable weight reduction, and SC (trial registration: German Clinical Trials Register DRKS00020249, Universal Trial Number U1111-1245-0273). In this paper, we report solely the results of the SC trial. More information regarding the other 2 trials can be found elsewhere [20-22]. The participants could participate in only 1 of the 3 RCTs. Nonetheless, in the IG of the SC trial, additional content of the other 2 health goals was optionally available.

### Ethical Considerations

This study followed the principles of the Declaration of Helsinki. It received a favorable opinion from the ethics commission of Albert-Ludwigs University, Medical Center, Freiburg (vote 237/19) on July 25, 2019. Informed consent of all the participants was acquired before their participation. Data protection for the study participants was guaranteed in accordance with the European General Data Protection Regulation. Staff members involved in the project were committed to such data protection through their institutions. All personal data concerning the participants were recorded and stored separately from pseudonymized research data by Vilua Healthcare GmbH. Vilua shared only pseudonymized or aggregated research data with Section of Health Care Research and Rehabilitation Research (SEVERA), Institute of Sport and Sport Science (IfSS), and Techniker Krankenkasse (TK). As compensation, the participants received shopping vouchers worth up to €30 (US $31.7) as well as additional discount vouchers (also refer to *Recruitment of Participants and Data Collection* section). The participants assigned to the CG were granted access to TK Smoking Cessation Coaching (TK-SCC) upon the completion of the study.

### Interventions

The 2 interventions used in the IG and CG, respectively, were both developed by a German statutory health insurance and had to be accessed via a web browser on the participants’ personal electronic devices. Participants in the IG had access to a version of TK-SCC [23] frozen at the end of December 2019. TK-SCC is an internet-based, interactive, and tailored program. It uses an abrupt cessation approach and incorporates various behavior change techniques (BCTs). The program’s preparation phase lasted between 1 and 14 days, depending on the individually determinable quit date, which could be specified by the participants according to their preferences. The preparation phase was followed by a 4-week maintenance phase. On the basis of the data from an integrated questionnaire, individualized activities with adapted intensities were automatically proposed by TK-SCC. The program displayed weekly planned activities, specific articles and advice, financial savings, and daily motivation tips on a personalized dashboard. Notifications depending on the progress of the participants provided feedback on the attained goals, motivated the participants to continue, or suggested specific barrier management. In case of inactivity, prompts to use the program were issued via email. Each participant had access to a personal profile page displaying an overview of their progress. In addition to the fully automated content of the program, TK-SCC included an offer for personal telephone counseling with specially trained counselors comprising up to 4 sessions.

In the CG, participants had access to a nontailored, noninteractive, and internet-based health program. It comprised evidence-based information divided into different lessons and advice on how to obtain smoking abstinence. It did not contain videos; the information provided was less detailed compared with the information provided to the IG, no feedback was given, and no prompts for using the program were issued to the participants in case of inactivity. No customized telephone counseling was offered, but the availability of a public offer for counseling was pointed out.

### Measures

**Figure 1:**
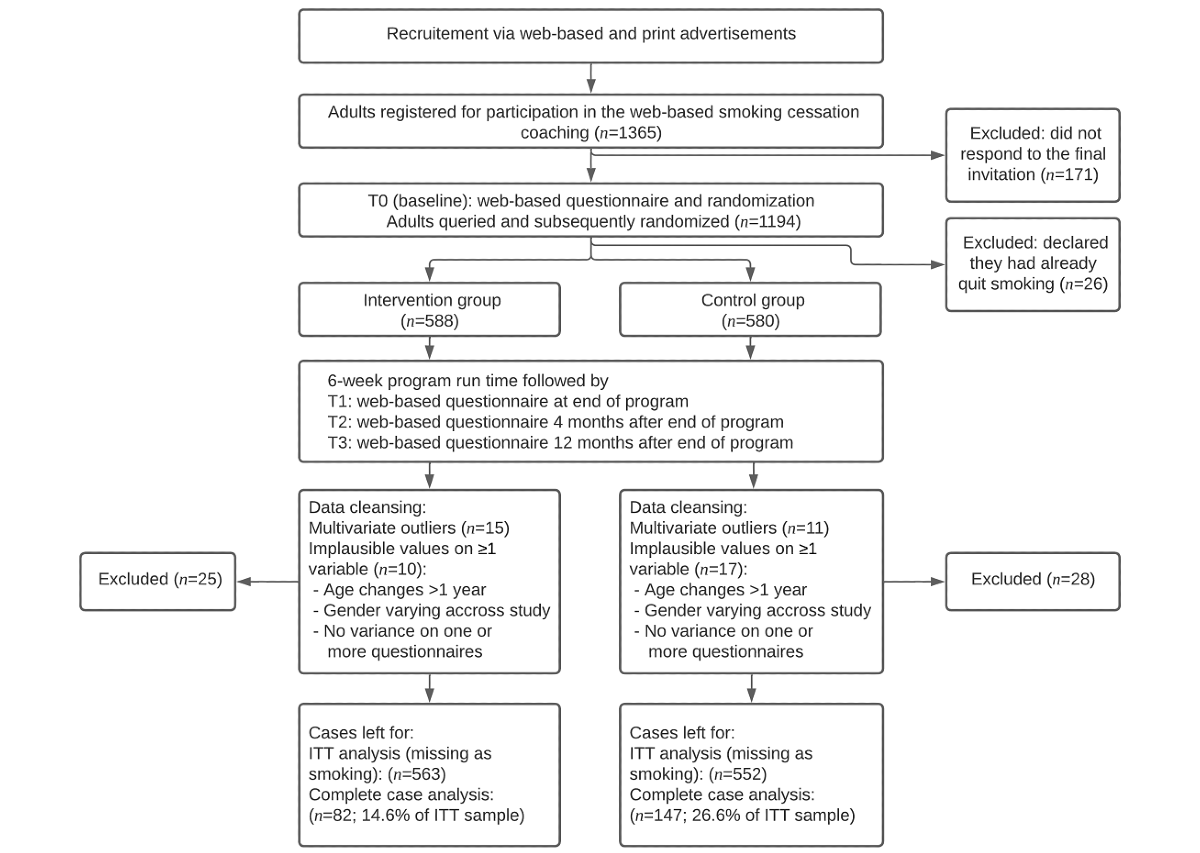
Flowchart of data collection, participants, and data cleansing. ITT: intention-to-treat; T1: postintervention time point; T2: 4-month follow-up; T3: 12-month follow-up.

Quantitative data (demographic information and self-reported outcomes) were collected via internet-based questionnaires at 4 measurement time points (Figure 1). The primary outcome was smoking abstinence in the last 30 days. We also gathered data on secondary outcome variables and confounder variables (Table 1).

**Table 1:** Overview of the instruments used to assess the primary outcome, secondary outcomes, and confounder variables.

Variables			Origin
**Primary outcome**			
	Smoking abstinence in the last 30 days: yes or no	Self-developed	
**Secondary outcomes**			
	Nicotine dependence	FTCD^a^ [24]	
	Health-related quality of life	SF-12^b^ [25]	
	Weight	IRES^c^ [26]	
	Self-efficacy in the process of behavioral change	Scale for the measurement of self-efficacy in the process of behavioral change in individuals who smoke [27]	
	Health-related goal intention	Goal intention [28]	
	Perceived goal attainment	Self-developed	
**Confounder variables**			
	Substance use (alcohol, cannabis, and hypnotics)	WHO^d^ ASSIST^e^ [29]	
	General state of health	BRAHMS^f^ [30]	
	Health impairments	KoMo^g^ [31]	
	Mental illness	PHQ-2^h^ [32] and an additional self-developed item	
	Social support for smoking cessation	PIQ-20^i^ [33]	
	A close acquaintance is smoking	Self-developed	
	Use of another SC^j^ program	Self-developed	

^a^FTCD: Fagerström Test for Cigarette Dependence.

^b^SF-12: Short Form 12 Health Survey.

^c^IRES: Indicators of Rehabilitation Status.

^d^WHO: World Health Organization.

^e^ASSIST: Alcohol, Smoking, and Substance Involvement Screening Test.

^f^BRAMHMS: Berlin Risk Appraisal and Health Motivation Study.

^g^KoMo: comorbidity score.

^h^PHQ-2: Patient Health Questionnaire-2.

^i^PIQ-20: Partner Interaction Questionnaire-20.

^j^SC: smoking cessation.

Furthermore, program use statistics (information on the frequency and duration of program log-ins, page views, and logged activities) were collected (IG only). In addition, we conducted qualitative telephone interviews with 15 (2.7%) of the 563 IG participants for an in-depth analysis of the participants’ experiences with the program.

### Recruitment of Participants and Data Collection

Sample size calculations were performed via G*Power (Universität Düsseldorf) [34], using an α error of .05, assuming a power of 0.80, and setting the expected effect sizes to a T3 abstinence rate of 15% in the IG and 2% in the CG. With an assumed dropout rate of 50% and a 10% buffer, our calculations resulted in a minimum overall sample size of 339 participants.

Between January and September 2020, we conducted recruitment campaigns in Germany using various media (Google marketing campaigns, communication channels of the Techniker health insurance fund and the University of Freiburg, flyers, local newspapers, radio channels, and print magazines). Data collection lasted until January 10, 2022. All adults who smoked, regardless of their health insurance supplier, were eligible for participation. The blinding of the participants could not be ensured, as both interventions were described in the study information. Prior to randomization, all participants were informed that those assigned to the CG would be granted access to TK-SCC upon the completion of the study (after the collection of T3 data). Blinded data analysis could not be achieved because the structure and content of the data indicated group allocation.

After completing the baseline questionnaire, participants were assigned to the IG and CG via permuted block randomization with block sizes of 4, 6, and 8 (Figure 1). The allocation of participants to the IG and CG was automated according to randomization lists generated by SEVERA using the RITA software (Universität zu Lübec), which were transmitted to the company contracted for data collection (Vilua GmbH). Participants who indicated at baseline (T0) that they had already quit smoking were excluded. Because of the initially high dropout rates following the postintervention time point (T1), we decided to change the system of incentivization midstudy from providing a single €25 (US $26.4) shopping voucher upon the completion of all 4 questionnaires to instead providing a €10 (US $10.6) voucher for each completed questionnaire from T1 to T3 as a reward. In addition, the participants received discount vouchers for GPS devices at T1 and the 4-month follow-up (T2). We sent up to 3 reminders via email if the participants did not complete a questionnaire.

### Data Cleansing, Missing Values, and Data Analysis

We cleaned the collected data to prevent the biasing of our results. During this process, we excluded cases with implausible values and multivariate outliers (through the comparison of the squared Mahalanobis distance of the items of each case with a chi-square distribution, exclusion of case if *P*<.001; refer to Figure 1).

All remaining randomized participants were included in the intention-to-treat (ITT) analysis, making the replacement of missing values necessary in case of the main outcome variable. Missing values were primarily generated when the participants either completely dropped out of the study following T0 or when they selectively missed 1 or 2 of the 3 consecutive questionnaires.

Missing data points were coded as smoking (*missing as smoking*). This procedure tends to lead to an underestimation of an existing effect, as it is unlikely that each missing participant was still smoking or relapsed. Therefore, ITT analyses with missing-as-smoking data replacement can be considered a conservative approach. Furthermore, as a means of sensitivity analysis for the main outcome variable, we conducted a complete case (CC) analysis, comprising only participants with a complete data set for all time points (regarding the primary outcome). This kind of analysis likely overestimates the real effect, as participants with complete data sets tend to be more motivated than the average.

Our primary outcome, *smoking abstinence in the last 30 days,* was constant at T0 because all the participants were smoking (n=1115, 100%). Therefore, statistical significance tests of changes over time were not possible for hypothesis 1. Instead, we used equivalence tests to compare the frequency of persons who stated to have become smoke free following the interventions with data from 2 Cochrane meta-analyses [13,14]. These meta-analyses reported success rates of 9% and 10.9% for SMS text messaging interventions and internet-based programs that were tailored or interactive or both, respectively. We assumed that the results of our interventions would be equivalent to those of these effective interventions and used a conservative threshold of +2% to −2% in the equivalence tests. To test hypothesis 2, we used binary logistic regression and included various confounder variables in the models (Table 1).

For the secondary outcomes (Table 1), replacement of missing values was not performed because of the high number of missing values. We used 2-factorial ANOVAs with repeated measures to investigate the effects upon these variables. Because this method of data analysis requires the participants to have a complete data set, only CC analyses could be conducted. When the Mauchly test of sphericity was significant, we used the Greenhouse-Geisser correction.

To test for a correlation between the use intensity and effect of TK-SCC (hypothesis 3), we assigned the IG participants to certain user types based on the number of their log-ins and the type of their use behavior. First, we made a rough distinction between 2 log-in groups: onetime users with only 1 log-in over the intervention period and multitime users with multiple log-ins over the intervention period. Following this, the multitime users were further subdivided into 3 classes by means of a latent class analysis. For this analysis, the dichotomized use of TK-SCC at the weekly level (0=no use and 1=use) was used to generate categorical indicator variables. The final model was selected based on the best model fit, which was determined using various parameters (eg, group size of the classes found, Bayes information criterion, and entropy). Finally, the following 4 log-in classes emerged: onetime users, rare users (who used the coaching only during the first week), half-time users (who ceased use after half of the intervention period), and constant users (who used TK-SCC throughout the intervention period). The latter case represents the use intended by the developers of the program (use per protocol). To test hypothesis 3, binary logistic regression with user types as the independent variable and smoking abstinence as the dependent variable was used.

In all cases of hypothesis testing, we controlled the familywise error rate using the Bonferroni-Holm method [35]. Data preparation and calculation of the scales were performed using R Statistics (version 4.1.3; R Foundation for Statistical Computing) and RStudio (version 2021.09.1; R Foundation), and equivalence tests were conducted using the *TOSTER package* [36]. For all other analyses, the SPSS Statistics software (version 28.0.0.0; IBM Corp) was used. This paper was prepared according to the CONSORT-EHEALTH (Consolidated Standards of Reporting Trials of Electronic and Mobile Health Applications and Online Telehealth) checklist [37].

## Results

### Sample Characteristics

The final sample for the ITT analysis (n=1115) consisted of 563 (50.49%) participants in the IG and 552 (49.51%) participants in the CG. The CC subsample (n=229) comprised 82 (35.8%) participants in the IG and 147 (64.2%) participants in the CG (Figure 1). Most (IG: 301/563, 53.5%; CG: 213/552, 38.6%) of the missing values originated when participants dropped out between T0 and T1, likely stemming from participants aborting their intervention or their quit attempt (Table 2). In the ITT sample, the mean age was 41.5 (SD 11.9) years, 71.5% (797/1115) of the participants were women, and 62.4% (696/1115) were in a partnership or married. Nearly all participants (1015/1115, 91.03%) had tried to quit smoking before, with 59.3% (661/1115) reporting 1 to 4 previous quit attempts and 317% (354/1115) reporting >5 attempts. At T0, the average score on the Fagerström Test for Cigarette Dependence (FTCD) was 4.8 (SD 2.4) in both groups, with 3 to 4 points indicating moderate physical dependence and 5 to 6 points indicating severe physical dependence. This is higher than the average score for individuals who smoke in Germany (mean 2.5-3.2) [38] but lower than the reported mean score for those seeking cessation support (mean 5.2-6.6) [39]. The vast majority of participants (1018/1115, 91.3%) had no previous experience with internet-based health coaching and predominantly used TK-SCC on a private smartphone (775/1115, 69.5%). The number of participants who indicated the use of additional SC coaching at T0 was different in both groups (Table 3). The use of additional coaching increased in both groups until T3, with the CG starting from a lower level at T0 and reaching a slightly higher level at T3 than the IG. Of the IG participants, 26.6% (93/349) used at least 1 activity of 1 of the 2 optional health goals (improving your physical fitness: 25/349, 7.2%; improving your weight: 31/349, 8.9%; and both optional goals: 37/349, 10.6%). Tables 3 and 4 provide a more detailed breakdown of the demographics and confounder variables in both groups.

**Table 2:** Missing data from internet-based questionnaires.

Missing data	Intervention group (n=563), n (%)	Control group (n=552), n (%)
Without missing questionnaires	82 (14.6)	147 (26.6)
Only T1^a^ questionnaire missing	43 (7.6)	15 (2.7)
Only T2^b^ questionnaire missing	8 (1.4)	19 (3.4)
Only T3^c^ questionnaire missing	30 (5.3)	40 (7.2)
T1 and T2 questionnaires missing	32 (5.7)	30 (5.4)
T1 and T3 questionnaires missing	24 (4.3)	23 (4.2)
T2 and T3 questionnaires missing	43 (7.6)	65 (11.8)
T1, T2, and T3 questionnaires missing	301 (53.5)	213 (38.6)

^a^T1: postintervention time point.

^b^T2: 4-month follow-up.

^c^T3: 12-month follow-up.

**Table 3:** Demographics of the participants in the IG^a^ and CG^b^.

Demographics			IG		CG
Age (years), mean (SD)			42.2 (11.9)		40.9 (11.8)
**Gender, n (%)**					
	Woman	408 (72.5)		389 (70.5)	
	Man	154 (27.4)		162 (29.3)	
	Diverse	1 (0.2)		1 (0.2)	
**Education, n (%)**					
	No graduation or another graduation	7 (1.2)		5 (0.9)	
	Primary or lower secondary school	47 (8.3)		53 (9.6)	
	Secondary school	197 (35)		186 (33.7)	
	Intermediate school or qualification for entry to higher education	80 (14.2)		77 (13.9)	
	High school graduation	232 (41.2)		231 (41.8)	
**Familial status, n (%)**					
	Single	124 (22)		142 (25.7)	
	Married or partnership	359 (63.8)		336 (60.9)	
Employment status (employed), n (%)			441 (78.5)		432 (78.3)
Insured with TK^c^ health insurance, n (%)			322 (57.2)		330 (59.8)
Prior experience with internet-based health coaching (yes), n (%)			10 (1.9)		7 (1.4)
**Previous attempts to stop smoking, n (%)**					
	Never	47 (8.3)		53 (9.6)	
	1-4	333 (59.2)		328 (59.4)	
	≥5	183 (32.5)		171 (31)	
Use of TK-SCC^d^ on a smartphone, n (%)			155 (72.4)		143 (66.5)
**Use of another SC^e^ program, n (%)**					
	Use of another SC program at T0^f^ (yes)	66 (11.7)		42 (7.6)	
	Use of another SC program between T0 and T1^g^ (yes)	122 (21.7)		124 (22.5)	
	Use of another SC program between T0 and T2^h^ (yes)	138 (24.6)		147 (26.7)	
	Use of another SC program between T0 and T3^i^ (yes)	157 (27.9)		161 (29.2)	

^a^IG: intervention group.

^b^CG: control group.

^c^TK: Techniker Krankenkasse.

^d^TK-SCC: Techniker Krankenkasse Smoking Cessation Coaching.

^e^SC: smoking cessation.

^f^T0: baseline.

^g^T1: postintervention time point.

^h^T2: 4-month follow-up.

^i^T3: 12-month follow-up.

**Table 4:** Confounder variables in IG^a^ and CG^b^ at T0^c,d^.

Confounder variables at T0		IG	CG
Alcohol consumption, mean (SD)		2.2 (1.2)	2.2 (1.3)
Cannabis consumption, mean (SD)		0.2 (0.8)	0.3 (0.8)
Cannabis consumption, median (IQR)		0.0 (0.0-0.0)	0.0 (0.0-0.0)
Hypnotics and sedative consumption, mean (SD)		0.2 (0.7)	0.2 (0.7)
Hypnotics and sedative consumption, median (IQR)		0.0 (0.0-0.0)	0.0 (0.0-0.0)
General state of health, mean (SD)		5.4 (2.0)	5.4 (2.2)
Mental illness: PHQ-2^e^, mean (SD)		2.0 (0.8)	2.1 (0.7)
KoMo^f^, mean (SD)		0.4 (0.5)	0.4 (0.5)
KoMo, median (IQR)		0.3 (0.0-0.6)	0.3 (0.0-0.6)
**Social support, mean (SD)**			
	PIQ^g^ negative	2.3 (0.7)	2.3 (0.8)
	PIQ positive	1.9 (0.7)	1.9 (0.6)
	PIQ saboteur	1.9 (0.6)	1.9 (0.6)
Smoking of an important acquaintance: yes, n (%)		491 (89.6)	488 (90.5)

^a^IG: intervention group.

^b^CG: control group.

^c^T0: baseline.

^d^Scale range: consumption of substances is rated on a scale ranging from 0 (“never”) to 4 ([almost] daily); general state of health is rated on a scale ranging from 0 (“far worse than others”) to 10 (“far better than others”); Patient Health Questionnaire–2 score ranges from 1 (“not at all”) to 4 (“nearly every day”); comorbidity score ranges from 0 (“no comorbidity”) to 10 (“high comorbidity”); and Partner Interaction Questionnaire–20 score ranges from 1 (“almost never”) to 4 (“almost all the time”).

^e^PHQ-2: Patient Health Questionnaire–2.

^f^KoMo: comorbidity score.

^g^PIQ: Partner Interaction Questionnaire–20.

### The Effects of the Interventions Over Time

At T1, 11.5% (65/563) of the participants in the IG had become smoke free (ITT, missing as smoking). This value slightly decreased until T2 (59/563, 10.5%) and increased again to 11.9% (67/563) at T3. In the CG, 6.2% (34/552) of the participants had become smoke free at T1, which slightly increased to 6.9% (38/552) at T2 and 8.2% (45/552) at T3. In case of the CC subsample, the proportion of participants stating to be smoke free in the IG was 57.3% (47/82) at T1, 43.9% (36/82) at T2, and 51.2% (42/82) at T3, compared with 12.9% (19/147) at T1, 19.7% (29/147) at T2, and 23.1% (34/147) at T3 in the CG (Table 5).

Regarding the proportion of participants who were smoke free and its change over time, it can be noted that in the IG, the value either remained relatively constant (ITT) or slightly decreased (CC), whereas in the CG, the value tended to increase over time, especially in the CC subsample. As a point of reference, we compared our results with the findings from a current Cochrane meta-analysis that reported a 9% abstinence rate 6 to 12 months following SMS text messaging interventions (and 6% for the comparator, minimal SC support) [13]. In addition, we compared our results with the findings from another Cochrane meta-analysis that reported a 10.9% abstinence rate at least 6 months after internet interventions which were tailored or interactive or both [14]. The ITT abstinence rates in our IG at T3 were slightly higher than those in the corresponding conditions of the meta-analyses. The equivalence test with +2% or –2% bounds was not significant, with a success rate of 9% (*z score*=0.64; *P*=.74). With a success rate of 10.9%, the test of equivalence (*z score*=−0.71; *P*=.24) was also not significant. These results indicate that the success rate in this study was statistically equivalent to the clinically relevant effect sizes in the 2 meta-analyses. Therefore, we conclude that hypothesis 1 is confirmed.

The secondary outcomes were interval-scaled variables, which made it possible to perform statistical significance tests on the intervention effects. These outcomes were tested in 2-factorial, repeated-measures ANOVA (Table 6).

**Table 5:** Descriptive data of the primary outcome (smoking abstinence) for ITT^a^ and CC^b^ analyses (n=1115).

Sample and primary outcome			T0^c^		T1^d^		T2^e^	T3^f^
**ITT sample (missing as smoking)**								
	**IG (n=563)**							
		Participants who were smoke free, n (%)	0 (0)	65 (11.5)		59 (10.5)		67 (11.9)
		Participants who were smoke free, 95% CI	—	8.9-14.2		7.9-13.0		9.2-14.6
	**CG (n=552)**							
		Participants who were smoke free, n (%)	0 (0)	34 (6.2)		38 (6.9)		45 (8.2)
		Participants who were smoke free, 95% CI	—	4.2-8.2		4.8-9.0		5.9-10.4
**CC subsample**								
	**IG (n=82)**							
		Participants who were smoke free, n (%)	0 (0)	47 (57.3)		36 (43.9)		42 (51.2)
		Participants who were smoke free, 95% CI	—	46.6-68.0		33.2-54.6		40.4-62.0
	**CG (n=147)**							
		Participants who were smoke free, n (%)	0 (0)	19 (12.9)		29 (19.7)		34 (23.1)
		Participants who were smoke free, 95% CI	—	7.5-18.3		13.3-26.2		16.3-29.9

^a^ITT: intention-to-treat.

^b^CC: complete case.

^c^T0: baseline.

^d^T1: postintervention time point.

^e^T2: 4-month follow-up.

^f^T3: 12-month follow-up

**Table 6:** The interventions’ effects for the IG^a^ and CG^b^ with time: results of the 2-factorial repeated-measures ANOVA for the secondary outcomes^c^.

Secondary outcomes (changes with measurement time points)	IG (n=82), n (%)	CG (n=147), n (%)	*F* test (*df*)	*P* value	Cohen *f*
Cigarette dependence (FTCD)^d,e^	25 (30.5)	98 (66.7)	20.699 (3, 363)	<.001	0.414
Quality of life	80 (97.6)	146 (99.3)	11.237 (2.86, 635.37)	<.001	0.225
Weight	77 (93.9)	141 (95.9)	4.109 (1.48, 319.68)	.03^f^	0.139
Self-efficacy	81 (98.8)	146 (99.3)	29.437 (2.67, 599.64)	<.001	0.362
Goal intention	82 (100)	146 (99.3)	6.496 (2.59, 585.71)	<.001	0.170
Satisfaction with goal attainment^g^	81 (98.8)	146 (99.3)	2.211 (1.85, 418.34)	.12	0.101

^a^IG: intervention group.

^b^CG: control group.

^c^All participants of the intention-to-treat sample were eligible, but the repeated-measures analyses could be carried out only for participants with complete data for a given outcome variable.

^d^FTCD: Fagerström Test for Cigarette Dependence.

^e^Only participants who resumed smoking completed the Fagerström Test for Cigarette Dependence.

^f^*P* value not significant following α error correction.

^g^Measured at the postintervention time point, 4-month follow-up, and 12-month follow-up.

Analyses could be performed only for participants with complete data for a given outcome variable. All measures showed statistically significant changes between T0 and T3, except for satisfaction with goal attainment (measured between T1 and T3; Table 6). With the exception of the difference in weight, all these differences remained significant after α error correction using the Bonferroni-Holm method [35]. The scores for cigarette dependence for participants who resumed smoking (FTCD) and the scores for goal intention decreased between T0 and T3, whereas the scores for quality of life, weight, and self-efficacy increased between T0 and T3. In case of the cigarette dependence scores, a large effect was demonstrated according to Cohen [40], whereas a medium-sized effect was found for self-efficacy. The other effects were small (Table 6).

### Differences Between the IG and CG

When examining the differences between the IG and CG with regard to the primary outcome (smoking abstinence), clear differences between the ITT sample (missing as smoking) and the CC sample emerged. In case of the ITT sample, after α error correction according to the Bonferroni-Holm method [35], a significant difference in favor of the IG with an odds ratio (OR) of 2.0 (99% CI 1.1-3.6; *P*=.002) could be detected only for T1. This was true for both the analysis with and the analysis without confounder variables (Table 7). In addition, we included gender as a covariate because of the higher number of women in our sample. The *P* value for this covariate was nonsignificant for all measurement time points (*P*>.05) and the ORs between the IG and CG did not change in a relevant manner when gender was added.

**Table 7:** Group differences for the primary outcome (smoking abstinence in the last 30 days). The variance explained (Nagelkerke R^2^); ORs^a^ between the intervention and control groups; and *P* values and the CIs at time points T1^b^, T2^c^, and T3^d^ are presented.

Time point, sample, and variable			Nagelkerke *R*^2^	OR (99% CI)	*P* value
**T1**					
	**ITT^e^ sample (missing as smoking)**				
		Without confounders	.020	2.0 (1.1-3.5)	.002
		Confounders included	.052	2.0 (1.1-3.6)	.002
	**CC^f^ sample**				
		Without confounders	.280	9.0 (4.7-17.3)	<.001
		Confounders included	.339	9.3 (3.7-23.5)	<.001
**T2**					
	**ITT sample (missing as smoking)**				
		Without confounders	.009	1.6 (0.9-2.8)	.03^g^
		Confounders included	.041	1.6 (0.9-2.8)	.03^g^
	**CC sample**				
		Without confounders	.090	3.2 (1.8-5.8)	<.001
		Confounders included	.168	2.9 (1.3-6.6)	.001
**T3**					
	**ITT sample (missing as smoking)**				
		Without confounders	.008	1.5 (0.9-2.6)	.04^g^
		Confounders included	.082	1.6 (0.9-2.7)	.02^g^
	**CC sample**				
		Without confounders	.107	3.5 (2.0-6.2)	<.001
		Confounders included	.213	3.4 (1.5-7.8)	<.001

^a^OR: odds ratio.

^b^T1: postintervention time point.

^c^T2: 4-month follow-up.

^d^T3: 12-month follow-up.

^e^ITT: intention-to-treat.

^f^CC: complex case.

^g^*P* value not significant following α error correction.

By contrast, for the CC sample, we found a significantly higher abstinence rate in the IG at all measurement time points, both before and after adjusting for confounders. At T1, the ORs were 9.0 (99% CI 4.7-17.3; *P*<.001; without covariates) and 9.3 (99% CI 3.7-23.5; *P*<.001; with covariates). At T2 to T3, ORs between 2.9 (99% CI 1.3-6.6; *P*=.001) and 3.5 (99% CI 2.0-6.2; *P*<.001) were detected (Table 7).

All these effects remained significant after α error correction. Thus, the analysis of the ITT sample and CC sample led to somewhat diverging results. However, because we had defined the results of the ITT analysis at T3 as the primary criterion for our conclusions, hypothesis 2 could not be confirmed.

In addition, we examined a number of secondary outcomes for an interaction effect between group and measurement time using 2-factorial repeated-measure ANOVAs. Following α error correction, the interaction effects between time and group remained significant for 2 of the 6 outcomes: self-efficacy (*P*<.001) and satisfaction with goal attainment (*P*=.002). There was an increase in self-efficacy in the IG at T1, followed by a slight decrease, whereas no significant increase was observed in the CG between T0 and T3. Likewise, in case of satisfaction with goal attainment, the IG had higher scores at T1, followed by a slight decrease over time, whereas in the CG, there was a slight increase following T1, although they did not reach the IG’s level at T3. In each case, the differences can be considered small effects [40]. No significant interaction effects were found for the other secondary outcomes (all *P*>.05; Table 8).

**Table 8:** Results of the 2-factorial repeated-measures ANOVAs for the secondary outcomes: interaction effects of time and group. All the participants of the ITT sample were eligible, but the analyses could be carried out only for participants with complete data for a given outcome variable.

Secondary outcomes (interaction effects of time and group)	IG^a^ (n=82), n (%)	CG^b^ (n=147), n (%)	*F* test (*df)*	*P* value	Cohen *f*
FTCD^c,d^	25 (30.5)	98 (66.7)	0.517 (3, 363)	.67	0.063
Quality of life	80 (97.6)	146 (99.3)	0.594 (2.84, 635.37)	.61	0.055
Weight	77 (93.9)	141 (95.9)	1.168 (1.48, 319.68)	.30	0.071
Self-efficacy	81 (98.8)	146 (99.3)	9.594 (2.67, 599.64)	<.001	.0207
Goal intention	82 (100)	146 (99.3)	2.735 (2.59, 585.71)	.05	0.110
Satisfaction with goal attainment^e^	81 (98.8)	146 (99.3)	6.475 (1.85, 418.34)	.002	0.170

^a^IG intervention group.

^b^CG: control group.

^c^FTCD: Fagerström Test for Cigarette Dependence.

^d^Only participants who resumed smoking completed the Fagerström Test for Cigarette Dependence (FTCD).

^e^Measurement available only from the postintervention time point to the 12-month follow-up.

### Relationship Between Use and Effect in the IG

To examine the association between TK-SCC use and effect (hypothesis 3), we conducted logistic regressions with user types as the independent variable and smoking abstinence as the dependent variable. The frequency and proportion of participants who were smoke free in the IG are listed according to user type in Table 9. The results of the logistic regressions with rare users as the reference category are presented in Table 10. Because cell sizes were small in some cases, we conducted this analysis only for the ITT sample.

We found statistically significant group differences between rare users and 2 of the other 3 user types: at T1, half-time users and, especially, constant users were more often smoke free than rare users (OR 5.3, 99% CI 1.5-19.6; *P*<.001; and OR 15.0, 99% CI 6.1-36.9; *P*<.001; respectively). At T2 and T3, only constant users differed significantly from rare users, with ORs of 8.3 (99% CI 3.3-20.9; *P*<.001) and 6.5 (99% CI 2.8-15.5; *P*<.001; Table 10). All these differences remained statistically significant following α error correction. By contrast, we could not find statistically significant differences between onetime users and rare users at any measurement time point. Therefore, hypothesis 3 was confirmed.

**Table 9:** Number and percentage of participants who were smoke free in the intervention group, grouped by use behavior (intention-to-treat sample, missing as smoking).

Log-in type	T1^a^, n (%)	T2^b^, n (%)	T3^c^, n (%)
One-time users (n=195)	8 (4.1)	15 (7.7)	17 (8.7)
Rare users (n=279)	18 (6.5)	18 (6.5)	24 (8.6)
Half-time users (n=26)	7 (26.9)	3 (11.5)	2 (7.7)
Constant users (n=63)	32 (50.8)	23 (36.5)	24 (38.1)

^a^T1: postintervention time point.

^b^T2: 4-month follow-up.

^c^T3: 12-month follow-up.

**Table 10:** The association between user type and smoking abstinence (intervention group only, intention-to-treat sample, missing as smoking): results of the logistic regressions with user types as the independent variable and smoking abstinence as the dependent variable. Rare users are the reference category.

Time point and log-in type		Nagelkerke *R*^2^	OR^a^ (99% CI)	*P* value
**T1^b^**				
	One-time users	.274	0.6 (0.2-1.9)	.27
	Half-time users	.274	5.3 (1.5-19.6)	<.001
	Constant users	.274	15.0 (6.1-36.9)	<.001
**T2^c^**				
	One-time users	.131	1.2 (0.5-)3.1	.60
	Half-time users	.131	1.9 (0.3-10.4)	.34
	Constant users	.131	8.3 (3.3-20.9)	<.001
**T3^d^**				
	One-time users	.113	1.0 (0.4-2.4)	.97
	Half-time users	.113	0.9 (0.1-6.4)	.87
	Constant users	.113	6.5 (2.8-15.5)	<.001

^a^OR: odds ratio.

^b^T1: postintervention time point.

^c^T2: 4-month follow-up.

^d^T3: 12-month follow-up.

## Discussion

### Principal Findings

We compared the effectiveness of a tailored and interactive eHealth intervention for SC with that of a noninteractive, nontailored, and information-only internet-based intervention.

In the IG, 11.9% (67/563) of the participants reported 30-day abstinence at 12 months. Although comparisons with different trials have to be made with caution, this abstinence rate was statistically equivalent to the average abstinence rates for similar interventions (9% and 10.9%) reported in 2 recent meta-analyses that we used as reference points [13,14]. These results were additionally supported by the analysis of secondary outcomes. However, the difference between the IG and CG was statistically significant only at T1 and not at T2 or T3. By contrast, sensitivity analyses with the CC sample showed significant differences between the IG and CG at both follow-ups, and an analysis of secondary outcomes revealed significant interaction effects of follow-up and group for 2 of the 6 measures (stronger increase in self-efficacy and higher values of goal satisfaction in the IG over time). However, because we defined the results of the ITT analysis (missing as smoking) at T3 as the primary criterion for our conclusions, hypothesis 2 could not be confirmed. Two possible explanations for this result seem plausible.

First, TK-SCC is not effective enough to achieve superiority compared with minimal SC support. In particular, the effects of tailoring and interactivity could be less important than previously theorized, or the implementation of these components could have been executed insufficiently. The same argument holds for the implementation of the BCTs used in TK-SCC. In this case, a critical examination and revision of TK-SCC would be required to improve its effectiveness. As a first step in this direction, we conducted a detailed qualitative analysis of the accounts of TK-SCC users [41]. In addition, measures could be taken to increase the use of the optional telephone counseling, which only 4.1% (23/563) of the IG participants used.

Second, the intervention used in the CG was more effective than minimal SC support. Indeed, 8.2% (45/552) of the participants in our CG were smoke free at T3. This abstinence rate is in line with the average effects of “other SC support alone,” “lower intensity SC support,” and “smartphone app” (with each having an 8% abstinence rate 6 to 12 months after the intervention), whereas “minimal SC support” lead to a 6% abstinence rate, on average, in the aforementioned meta-analysis [13]. One of the reasons for this outcome could be a cointervention bias [42]. When asked about having used or using an additional SC intervention, at T0, a total of 7.6% (42/552) of the participants in the CG replied “yes.” This percentage increased to 29.2% (161/552) for the period from T0 to T3. Unfortunately, owing to a large amount of missing data (70.2% [395/563] to 73.5% [414/563] in the IG), we could not use this as a covariate‬. However, because almost one-third (161/552, 29.2%) of the participants in the CG who answered this item indicated having used another SC intervention, it seems plausible that a relevant proportion of the participants in our “minimal SC support” condition did receive a more effective treatment than planned. This may have contributed to the unexpectedly good performance of our CG and our inability to confirm the second hypothesis.

In addition to an intervention effect and a difference between the groups, we predicted that a more intense use of TK-SCC would be positively related to a higher proportion of participants who successfully quit smoking. After identifying 4 user groups based on their use behavior, at the end of treatment, half-time users were more successful than participants who used TK-SCC less frequently; however, this effect did not last over time. Nevertheless, constant use (the use intended by the program developers) was strongly associated with smoking abstinence even 1 year after the end of treatment.

### Strengths and Limitations

We conducted a study with a thorough methodology (with implementation as an RCT, the coding of missing participants as participants who still smoke, a long period between the end of program and the last measurement point, the incorporation of various confounders, and α error correction for multiple testing). Furthermore, we were able to recruit a large sample of participants via various recruitment channels.

The limitations of our work are the lack of blinding of the participants; the considerable dropout rate (albeit this is common for internet-based interventions [16]; the retention rate could probably have been increased with higher incentives [43]); and the possibility of cointervention bias, which we were unable to prevent in the context of our study. Moreover, cell sizes for some log-in types were small, and the relationship we found between use intensity and effect was correlative and not causal. Two interpretations seem plausible: more regular use of TK-SCC results in long-term effects or less successful users stop using TK-SCC at an earlier stage. It seems likely that both explanations play their role. The results of our study may also have been affected by the COVID-19 pandemic and the ensuing restrictions and countermeasures in Germany [44], but this should have affected both groups to the same degree.

When planning a comparable study, future experimental designs should acknowledge the possibility that a relevant proportion of the participants will use additional eHealth interventions, which could complicate proving an existing intervention effect. We have 2 suggestions regarding this concern. First, the use of additional SC interventions should be systematically assessed and considered as a covariate. Second, the handling of participants with missing data as participants who still smoke (*missing as smoking*) is commonly used in many studies as a conservative method. However, this possibly hinders the ability to identify statistically significant group differences. This problem could be alleviated by minimizing dropout, for instance, by means of higher incentives.

### Comparison With Prior Work

The effects in our IG were statistically equivalent to the average effects of SMS text messaging interventions and internet-based programs that were either tailored, interactive, or both, as reported in 2 meta-analyses (9% and 10.9% smoke free after 6 to 12 months, respectively [13,14]). Therefore, our results confirm prior findings about the effectiveness of eHealth SC interventions [12,13]. Although the evidence concerning the effects of tailoring and interactivity in SC interventions is mixed, the combination of both techniques was previously found to be superior to other SC interventions [14]. This could not be confirmed by our results. However, our findings are in line with those of other studies reporting an association between the amount of use and effect of SC interventions [17,18].

### Conclusions

Our study adds to the existing evidence that eHealth SC interventions using BCTs, interactivity, and tailoring can be effective tools for increasing the quit rates of individuals who smoke, but superiority compared with a less intensive intervention could not be proven in the long term. Insufficient implementation of the techniques used in TK-SCC (interactivity, tailoring, or the used BCTs) as well as cotreatment bias could explain this outcome. Further analysis of the implementation of the techniques used in TK-SCC, possibly succeeded by program revision, seems promising. However, a more intense use of TK-SCC was positively related to a higher number of successful quitting attempts. Therefore, our results support the theory that additional efforts to keep the users of eHealth SC programs engaged might contribute to improving the effectiveness of these interventions. A suggested strategy could be the implementation of gamification [45].

## Appendix

Multimedia Appendix 1 CONSORT EHEALTH checklist (V 1.6.1).

## Abbreviations

BCT: behavior change technique
CC: complete case
CG: control group
CONSORT-EHEALTH: Consolidated Standards of Reporting Trials of Electronic and Mobile Health Applications and Online Telehealth
FTCD: Fagerström Test for Cigarette Dependence
IfSS: Institute of Sport and Sport Science
IG: intervention group
ITT: intention-to-treat
OR: odds ratio
RCT: randomized controlled trial
SC: smoking cessation
SEVERA: Section of Health Care Research and Rehabilitation Research
TK: Techniker Krankenkasse
TK-SCC: Techniker Krankenkasse Smoking Cessation Coaching

## Data Availability

The data sets generated or analyzed during this study are available from the corresponding author upon reasonable request. The prerequisite for this is that the anonymity rules promised to the participants are adhered to (eg, no publication of data that allows drawing conclusions about the identity of a person).
